# Supplementary material for: SSAM-lite: A Light-Weight Web App for Rapid Analysis of Spatially Resolved Transcriptomics Data
Source: Front Genet. 2022 Feb 28;13:785877. doi: 10.3389/fgene.2022.785877 (PMC8918671; doi:10.3389/fgene.2022.785877)
Supplement: Supplementary file 1 [file DataSheet1.pdf]

Supplementary Material

Supplementary Information

Differences between SSAM and SSAM-lite

Extended Benchmarking

Supplementary Figures

Supplementary Figure 1. Effects of the expression threshold and bandwidth parameters on the cell-type map.

Supplementary Figure 2. SSAM-lite approximates a naive KDE.

Supplementary Figure 3. SSAM-lite KDE implementation outperforms previous versions implemented in SSAM.

Supplementary Figure 4. Regional cell-type proportion estimates.

Supplementary Figure 5. Custom color palettes and cell-type highlighting.

Supplementary Figure 6. SSAM-lite runtime and memory usage scale linearly with dataset size.

1 Differences between SSAM and SSAM-lite

In order to improve the simplicity, usability and performance of SSAM-lite, we implemented certain heuristics and changed the feature set of SSAM-lite compared to SSAM's guided mode. This allowed for the implementation of a cleaner graphical user interface and simplified the parameter definitions. The differences are described in the following text.

1.1 KDE heuristics

As default, the original SSAM package utilizes the sklearn implementation of KDE in its *run_kde* function. In addition, it also has a *fast_kde* function which employs a heuristic for producing the KDE. Instead of computing an activation value for every single pixel in the pixel matrix, it only visits pixels that are close to the input coordinate points and updates surrounding pixels up to a distance where activation is considered negligible (as the Gaussian curve decays when moving away from the mean).

The differences between the SSAM-lite KDE implementation to that of SSAMs *fast_kde* stems from the way local pixels are updated in the pixel matrix. SSAM applies the Gaussian KDE to a small cutout of three bandwidths centered at the molecule coordinate only, whereas SSAM-lite first assigns each molecule coordinate in the pixel space and then projects a pre-calculated Gaussian kernel template onto the pixel space. This results in performance optimizations as the Gaussian kernel has to only be generated once, and we can directly obtain the results of the KDE in the pixel space (Figure S2). However, the output of SSAM-lite's KDE can deviate slightly from SSAM's original output when both bandwidth and resolution are low, although we believe these differences are negligible (Figure S2).

1.2 Per gene expression threshold

SSAM implements a per gene expression threshold in addition to the total expression threshold. We found that the total expression threshold always dominated over the per gene expression threshold, so removing it has negligible effect and allows us to simplify SSAM-lite with fewer parameters.

1.3 Automated scaling of coordinates to pixel matrix size

Other than the original SSAM, SSAM-lite includes a subroutine that automatically scales the input coordinates to fill out the complete pixel matrix and determines the width-height ratio from the coordinate input. This leads to optimal memory usage and direct control of the vector field size in pixels, whereas for SSAM, the user must provide the (metric) sample height and width externally and then define the shape of the pixel matrix through a scaling factor that determines the sampling distance. Beyond optimized memory consumption, this eliminates an additional parameter and allows for a simpler interface.

1.4 Area-based structure removal

The original SSAM implementation includes a function to remove small structures from the output cell-type map by: (i) removing structures ('blobs') with a pixel count below a user-defined threshold; and (ii) Filling empty blobs below a certain pixel count in the cell-type map. These features were omitted for SSAM-lite's initial release as we did not consider them elementary, and including them

would have complicated the interface. However, we're considering including them in a future release.

1.5 Correlation-based cutoff

The original SSAM algorithm provides a threshold for a minimal correlation before a pixel is classified, whereas SSAM-lite classifies all pixels above the expression threshold. We did not implement this feature because out of the two thresholding concepts, the pixel matrix norm threshold (which is used in SSAM-lite) seemed more intuitive and in our experience gives a good estimate for local cell existence.

1.6 Input and output masks

The original SSAM can make use of a user-provided input and output masks, which restricts the data processing to certain parts of the image. This feature does not affect the computation of the guided mode analysis. The mask must be computed by an external tool and requires careful fitting to the output cell-type map, which is why we decided against including it in our light-weight tool.

2 Extended Benchmarking

2.1 Low end hardware

To demonstrate the algorithm's ability to scale to modest hardware, we analyzed the mouse SSp osmFISH dataset by Codeluppi et al on a Lenovo b570e laptop and a 2017 Samsung S8+ Android 9 smartphone. The SSAM-lite algorithm was run with a parameter setting of bandwidth=5, pixel width=500 and threshold=2. The runtime and memory footprint of the function *runKDE* was monitored using the DevTools performance monitoring tool of Chrome v96. The procedure was repeated three times per test.

The Lenovo Laptop was equipped with a dual-core 2.20 GHz Intel CPU, 4GB of RAM, running a Windows 10 operating system and a Chrome v96 browser. *RunKDE* averaged at 36.9 seconds (32.41, 43.53, 43.86) with a standard deviation of 6.51 seconds. The heap memory footprint was 258 +/- 1.2 Mb (257, 257, 259).

The Samsung S8+ was equipped with an octa-core CPU (4x2.3 GHz Mongoose M2 & 4x1.7 GHz Cortex-A53), 4GB RAM, running the Android 9 operating system and the mobile version of Chrome v96. The benchmarking procedure was the same as described above, but using a remote USB-DevTools connection to a desktop browser for data recording. This time, *runKDE* averaged at 41.76 seconds (41.36, 37.34, 47.18) with a standard deviation of 4.03 seconds. The heap memory footprint was 255 +/- 1.2 Mb (256, 255, 253).

2.2 SSAM-lite KDE heuristic performance increase

To demonstrate that our new implementation of KDE is substantially faster than the implementation in SSAM, we implemented SSAM-lite's heuristic in python and compared it to the naive, C-based KDE function *run_kde* implemented as default in the SSAM package, and to a python version SSAM's *fast_kde* algorithm.

Using a simulated dataset of random coordinates on a square patch, we determined the resource consumption characteristics of the different KDE implementations by varying the ‘cell-type map width’, ‘n_coordinates’ and ‘kernel bandwidth’ parameters. The runtime of the naive SSAM *run_kde* proved the slowest, typically showing a 100-fold slower runtime increase compared to the SSAM *fast_kde* heuristic. SSAM-lite’s added heuristic could outperform SSAM’s *fast_kde* by 10-fold in terms of runtime. The runtime complexity of SSAM’s default *run_kde* algorithm scaled quadratically with respect to cell-type map width (or linear with cell-type map pixel count), whereas the SSAM *fast_kde* and the SSAM-lite heuristic implementation show a constant runtime for all cell-type map sizes (Figure S3).

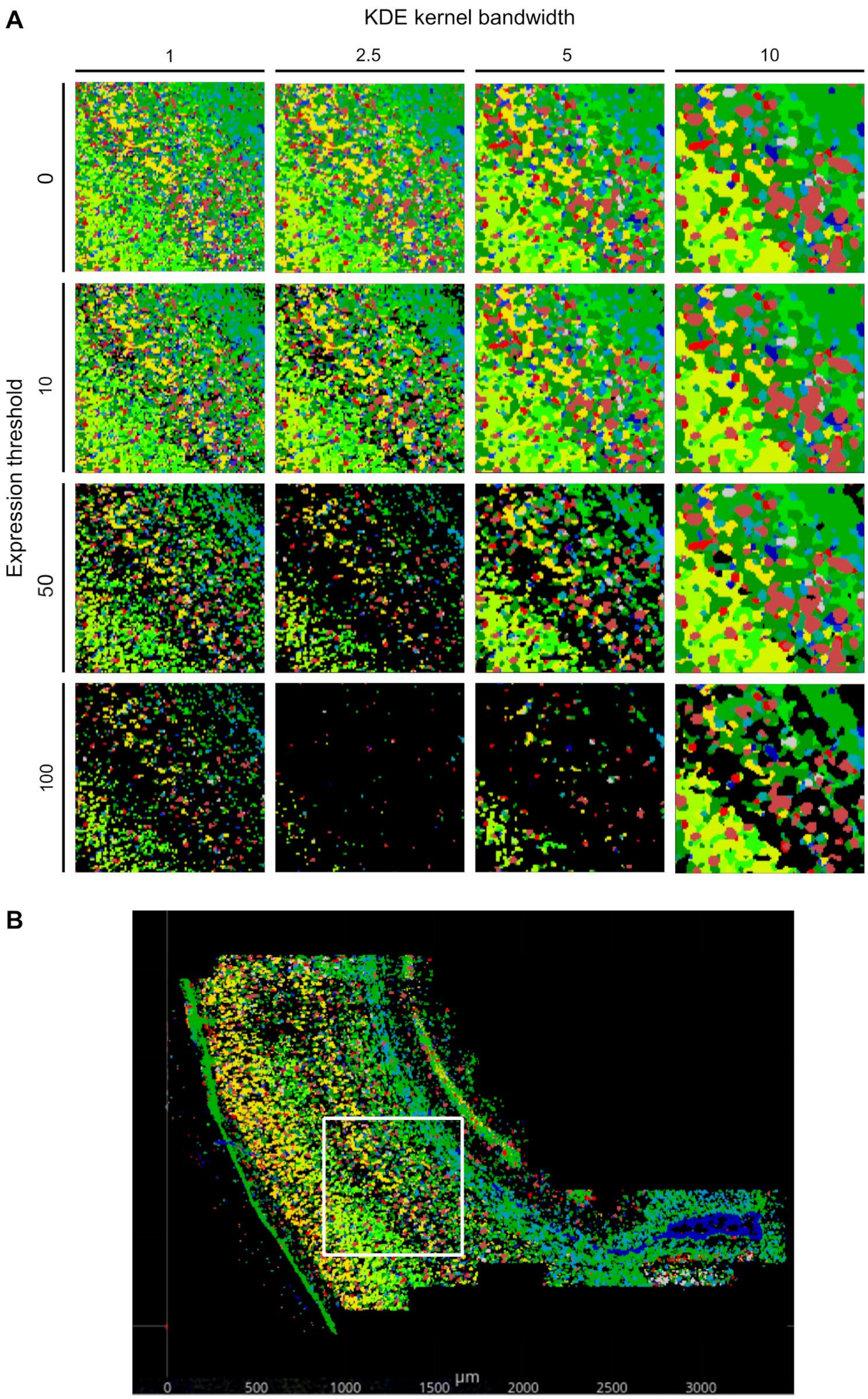

Supplementary Figure 1. Effects of the expression threshold and bandwidth parameters on the cell-type map. The entire SSAM-lite pipeline with different input parameters (A) was applied to the square subsection of the osmFISH mouse SSp dataset highlighted in (B). (A) Shows the output cell-type maps for different combinations of expression thresholds (0, 10, 50, 100) as rows and bandwidth parameters (0, 2.5, 5, 10) as columns. The effect of the bandwidth and expression threshold parameters are discussed in the Methods section.

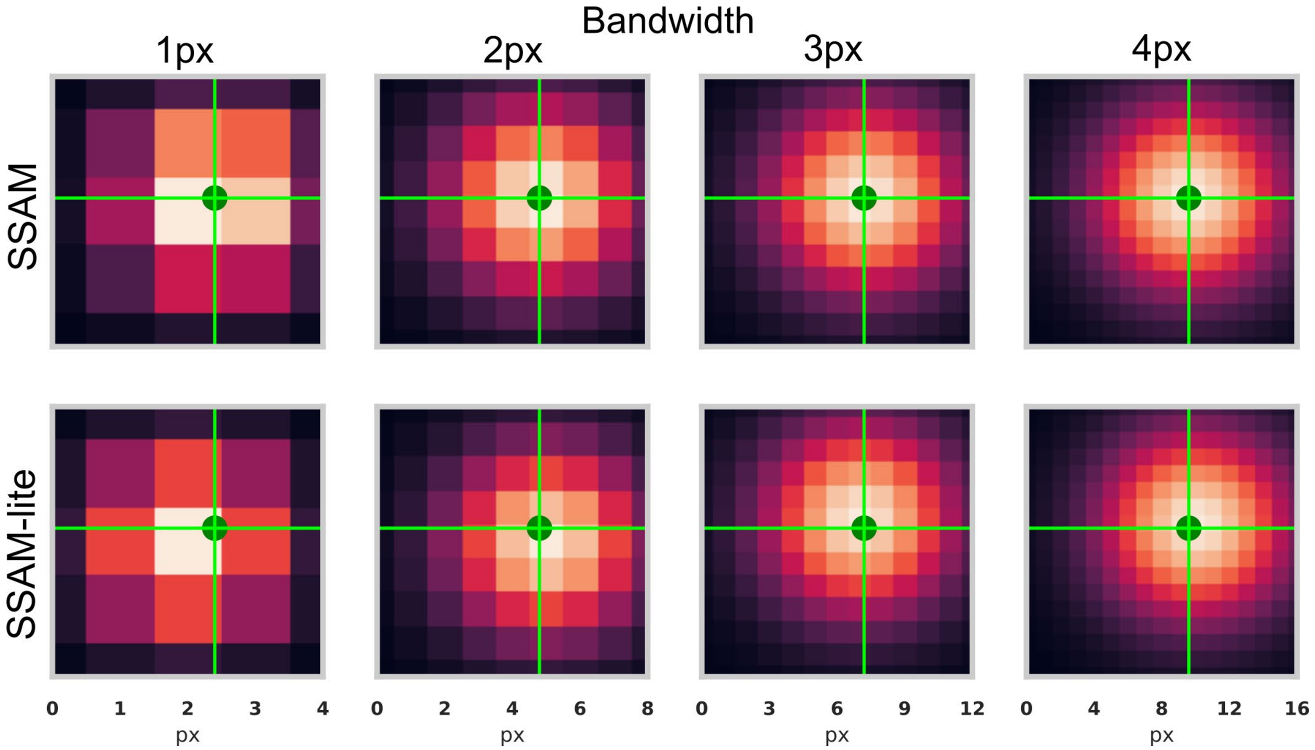

Supplementary Figure 2. SSAM-lite approximates a naive KDE. Since SSAM-lite pre-computes a template Gaussian kernel, it cannot model the fact the input coordinate is not centered on the pixel. The naive KDE however can adapt its Gaussian approximation to be slightly asymmetric and lean to the upper right. This divergence becomes more and more negligible when the resolution increases. Shown are the results of the KDE calculated by SSAM (top panels), and SSAM-lite (bottom panels), for different bandwidths for a given mRNA spot, shown as a green dot, with its x- and y-location highlighted by green lines.

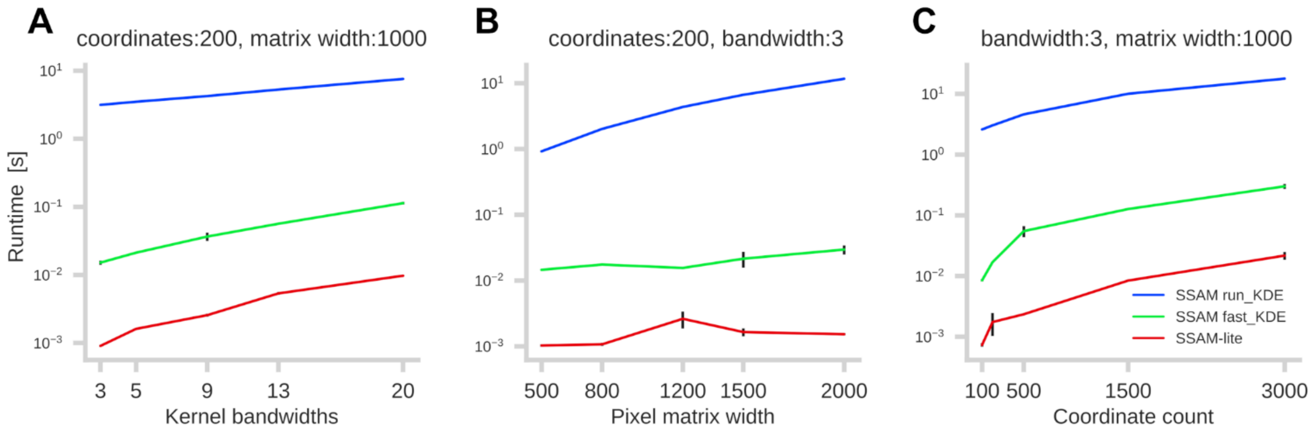

Supplementary Figure 3. SSAM-lite KDE implementation outperforms previous versions implemented in SSAM. In order to compare the performance of the SSAM-lite implementation of the KDE algorithm we compared the default KDE algorithm in SSAM, *run_KDE*, to the heuristic version implemented in SSAM, *fast_KDE*, and the SSAM-lite heuristic implementation. Both the *fast_KDE* and SSAM-lite KDE were re-implemented in python. Square pixel matrices of different widths were created and the KDE functions were called to transfer a range of randomly generated coordinates onto the matrix using different bandwidths. A range of parameters for bandwidths (A), matrix width (B) and coordinate count (B) were tested, while each of the other two parameters were kept constant. Each run was repeated three times, with the plot displaying the mean of the outputs, and the whiskers showing the standard deviation. SSAM-lite KDE was the fastest in all of the set-up, outperforming SSAM's *fast_KDE* by a factor 10. SSAM's *run_KDE* consumed the most time, being around 1000 times slower than SSAM-lite KDE.

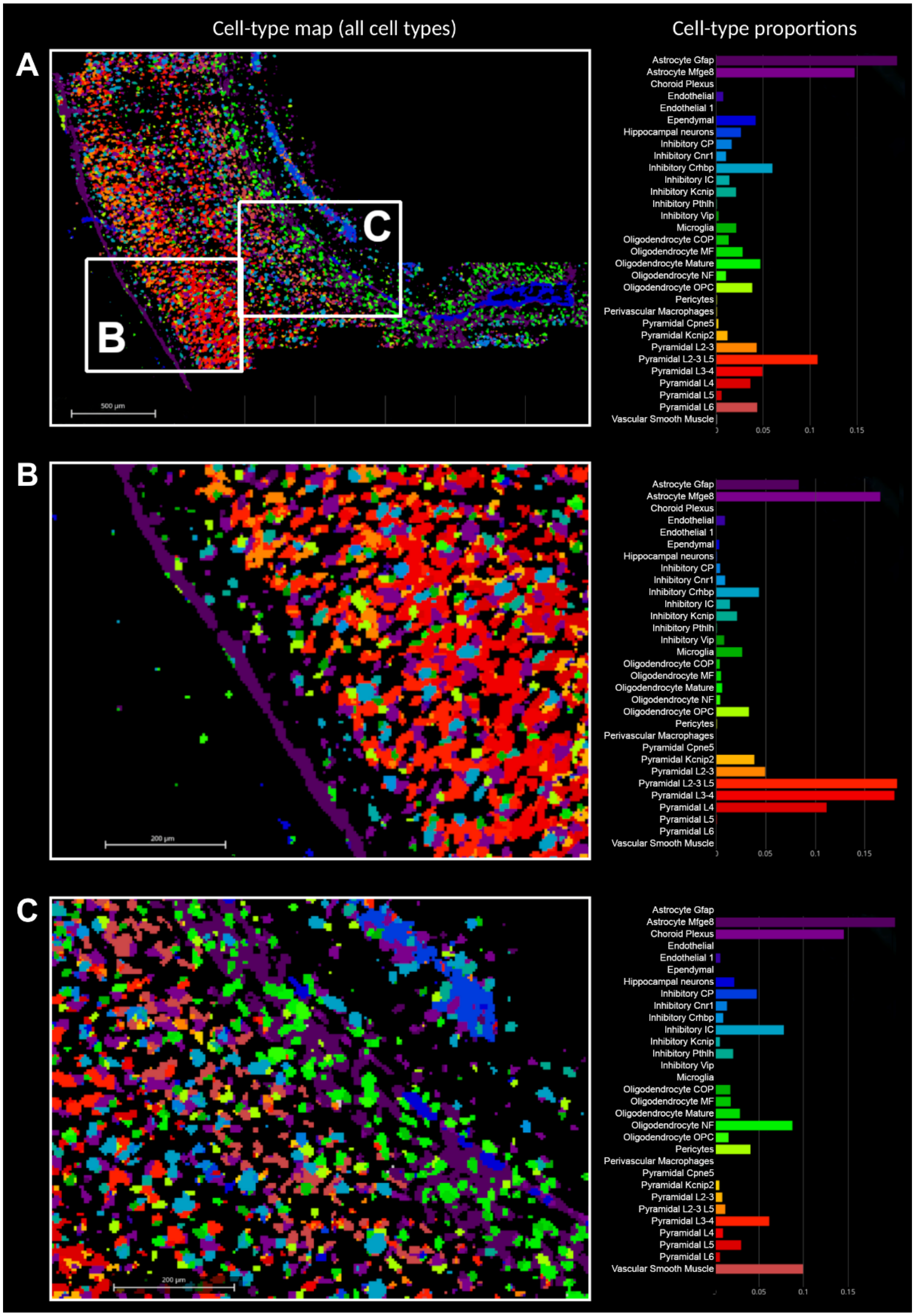

Supplementary Figure 4. Regional cell-type proportion estimates. Cell-type map and relative cell-type proportion estimates in the (A) entire mouse SSp, (B) zoom-in of the pia and cortex layers 1-4, and (C) zoom-in of cortex layers 4-6 and the hippocampus. Panel B shows enrichment of L2-3, L2-3 L5, L3-4 and L4 pyramidal cell types, and panel C shows enrichment of L2-3 L5, L4, and L6 cell types.

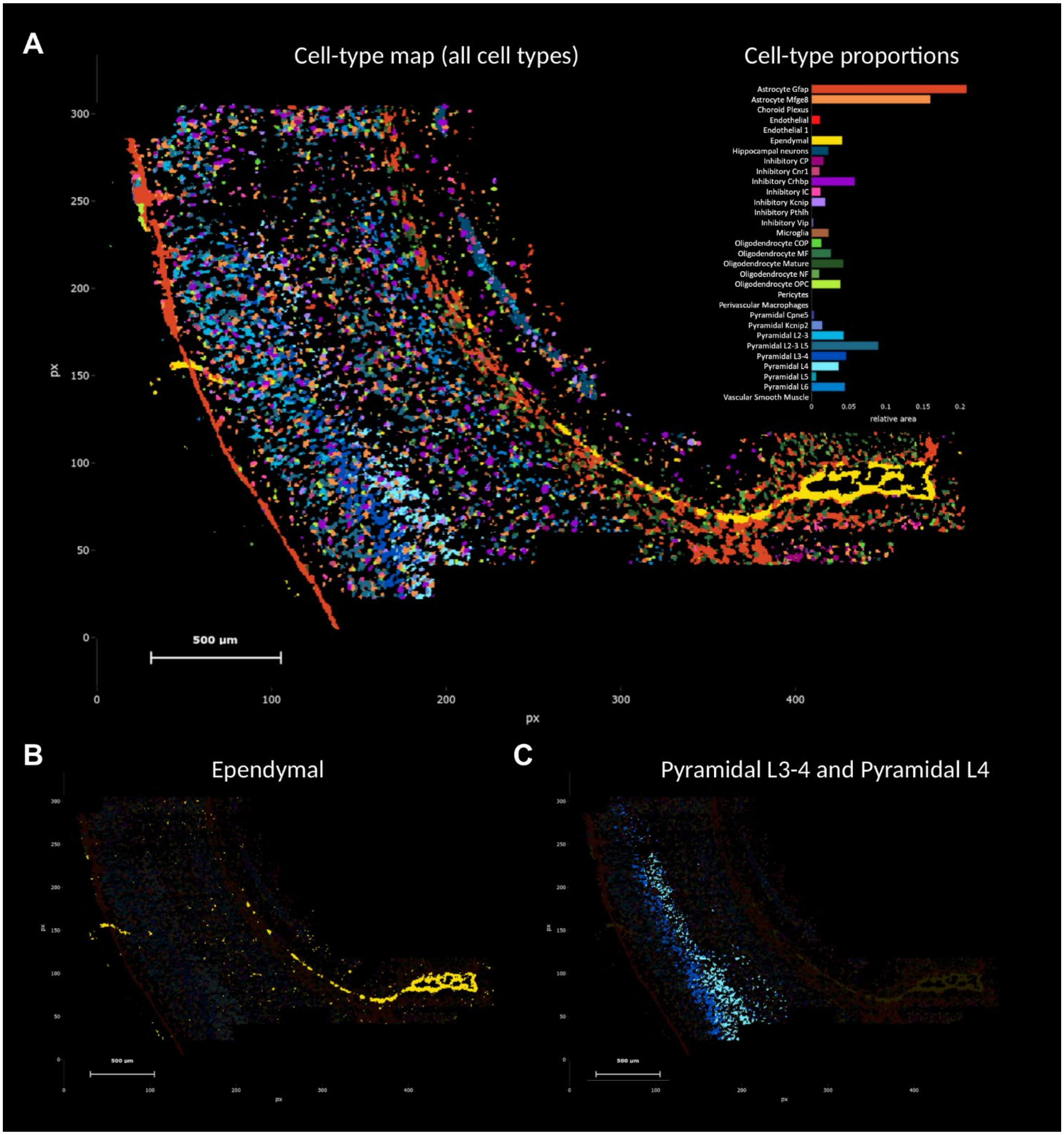

Supplementary Figure 5. Custom color palettes and cell-type highlighting. Cell-type map of (A) all cell types in the mouse SSp, (B) only ependymal, (C) and pyramidal L3-4 and pyramidal L4 cell-types. The color palette shows cell types rendered using the same colors as in Codeluppi et al (https://doi.org/10.1038/s41592-018-0175-z).

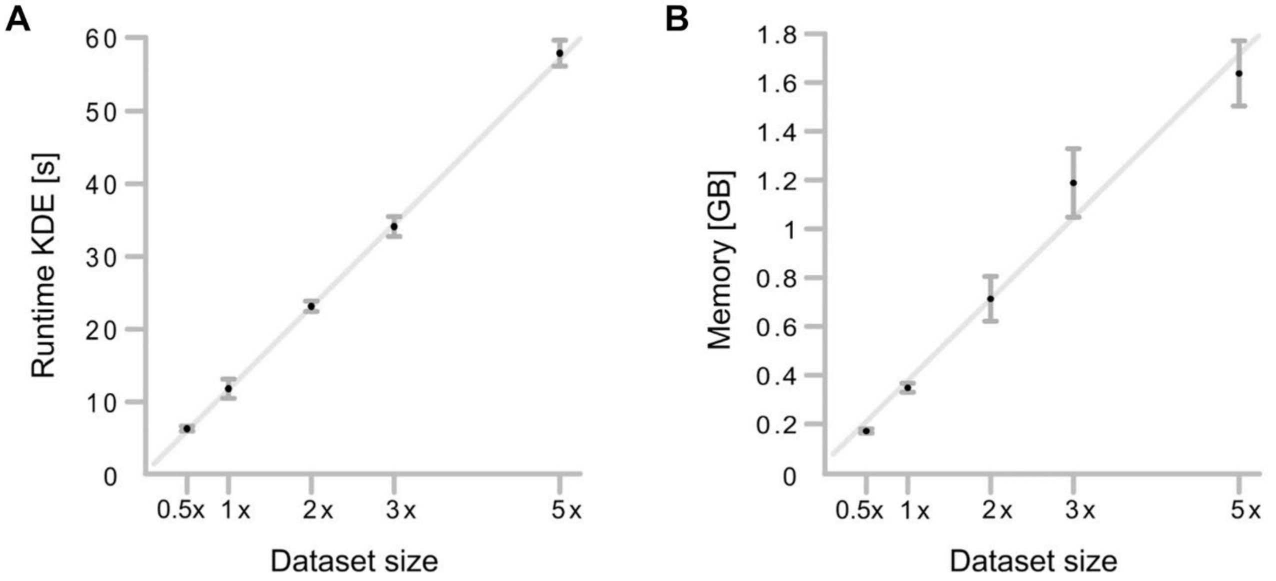

Supplementary Figure 6. SSAM-lite runtime and memory usage scale linearly with dataset size. The simulation was based on the mouse SSP osmFISH dataset which has 1.8 million mRNA spots. (A) Computation time for the KDE step scales linearly with data set size ($R^2 = 0.9996$, F-value=9445 and p-value < 2.41e-6). (B) Maximum RAM consumption scales linearly with dataset size ($R^2=0.9738$, F-value=149.9 and p-value < 1.18e-3). The SSP data set was down-sampled by 50% and up-scaled by a maximum factor of 5. Black dots are mean values. Error bars are standard deviations. (n=3). Grey line is a linear fit. The 0.5x dataset took on an average 6.32 seconds to run with a memory footprint of 171 MB. In comparison, the whole dataset took on average 11.81 seconds with a memory footprint of 343 MB. Whereas the largest 5x dataset on average took 57 seconds with a memory footprint of 1.6 GB. These results suggest that there is a linear increase of memory and CPU requirements with an increasing number of mRNA molecules profiled in a single experiment.
